# Modernising Hip Fracture Anaesthesia

**DOI:** 10.2174/1874325001711011190

**Published:** 2017-10-31

**Authors:** Hannah Dawe

**Affiliations:** St. Georges Hospital, Tooting, SW170QT, London, UK

**Keywords:** Hip fracture, NHFS, Spinal, ASAP, Transfusion

## Abstract

Hip fracture carries a 30-day mortality of around 8% in the United Kingdom. This figure has remained relatively unchanged despite modern developments in anaesthetic technique. These range from improvements in perioperative analgesia and mortality scoring systems, changes to intra-operative anaesthetic technique and strategies to reduce the requirement for blood transfusion. In this article, we review the current literature on the perioperative management of patients undergoing hip fracture surgery including some of the current controversies.

## 1 INTRODUCTION

Patients suffering a hip fracture are usually elderly, with a median age of 83 years [1], and co-existing chronic illnesses. Around 30% of patients with hip fracture also suffer from cognitive dysfunction and the requirement for urgent surgery in this patient group can be challenging to the anaesthetist [2]. It is well recognised that unnecessary delay to operative fixation of hip fractures is associated with increased mortality [3]. Therefore, the aim of anaesthetic perioperative management is to investigate and ‘normalise’ the patient within the time constraints, in order to provide standardised care, tailored to individual patient needs. This is done in conjunction with the Orthopaedic and Orthogeriatric teams.

In 2007, the British Orthopaedic Association and British Geriatrics Society published their ‘Blue book’ of standards for hip fracture care in the United Kingdom [4]. These standards are recorded by the UK National Hip Fracture Database (NHFD) which now audits numerous elements of hip fracture care allowing comparison of individual hospitals’ outcomes [5]. In 2012, the NHFD was amended to capture anaesthetic details. This data has been collated to provide insights into the variety of different anaesthetic techniques used in this challenging patient group.

The anaesthetist’s role may be very broad in hip fracture care, and is not restricted to the provision of anaesthesia during surgery. Pre-operatively the anaesthetist may be involved in providing analgesia in the form of a peripheral nerve block, ‘normalisation’ of the patient and providing information on certain risks of anaesthesia. Intra-operatively there remains significant debate regarding the most effective anaesthetic techniques, with a particular emphasis now being placed on avoiding intra-operative hypotension [1]. Post-operative anaemia may adversely affect patient outcomes and careful consideration should be given to peri-operative blood transfusion [6-8]

There are three UK national guidelines concerning the anaesthetic perioperative management of hip fracture [9-11]. The most relevant to the anaesthetist are ‘The management of proximal femoral fracture, 2011’, from the Association of Anaesthetists of Great Britain and Ireland (AAGBI), due to be updated in 2016 [11]. These provide a framework for hip fracture care although many of the elements of anaesthesia for hip fracture remain controversial.

## 2 PRE-OPERATIVE MANAGEMENT

### 2.1 Pre-Operative Analgesia

Hip fractures are painful, and providing analgesia for these patients whilst they await definitive surgery is paramount. Pain should be assessed regularly and adequate analgesia should be provided as soon as possible [9], including in the pre-hospital setting [10].

Both opioid analgesics and under-treatment of pain can have unwanted side effects including perioperative delirium [12]. This places the patient at further risk of harm, increases rates of perioperative mortality and impairs the ability of patients to be involved in decision-making regarding their care.

Patients with cognitive dysfunction, such as dementia may receive up to a third less morphine than those without cognitive impairment, suggesting their pain is underestimated and undertreated [13].

Regional techniques may be particularly useful in this high risk group.

Modern developments in pre-operative hip fracture care attempt to provide dynamic and static analgesia whilst minimising the complications associated with opioid usage.

Formal analgesia protocols are recommended, guided by regular assessment of the patient’s pain score. Where no contraindications exist, this multimodal approach consists of regular paracetamol, cautious use of opioids and considering peripheral nerve blockade for every patient. Non-steroidal drugs are avoided due to their adverse effects [9, 11].

A variety of peripheral blocks can be performed; most commonly fascia iliaca compartment blocks or femoral nerve blocks. These can be performed by a trained member of staff as soon as the diagnosis of hip fracture is confirmed. Such blocks have been demonstrated to provide superior analgesia compared to opioids and also to reduce opioid consumption [14-16].

Both fascia iliaca compartment blocks and femoral nerve blocks can be performed as either a ‘single shot’ or combined with a catheter through which local anaesthetic can be infused continually. The latter has the potential to allow constant analgesia throughout the entire perioperative period, with the catheter being removed postoperatively once the patient is comfortable. However, the indwelling catheter may migrate or become inadvertently removed (1.4%) as well as increasing the risk of infection (local infection rate 0-3-2%) compared to a single shot block [17].

Patients who receive a one shot block will initially have good analgesia, but as the block is of limited duration, patients may still require opioid medication whilst awaiting their definitive surgery [18].

Fascia iliaca blocks may be performed using either a landmark technique, or under ultrasound guidance [19]. Both are effective in providing acute pain relief, although ultrasound guidance has been shown to be superior [20]. Fascia iliaca ‘compartment’ blocks are so-called as they use large volumes of local anaesthetic, usually 30-40 ml [21, 22], which spreads under the fascia iliaca and blocks pain transmission in the femoral and lateral cutaneous nerves, possibly extending to also cover the obturator nerve and lumbar plexus [22].

Using the landmark technique, the compartment is located clinically by feeling two ‘pops’ as the needle passes through the fascial planes of the tensor fascia lata and fascia iliaca. Ultrasound guidance allows the fascia iliaca to be identified and local anaesthetic injected into the compartment under direct vision.

Absolute contraindications are allergy to local anesthetics and local skin infections. Relative contraindications include anticoagulation, but the risks and benefits must be considered for individual patients.

Potentially serious complications which can occur include intravascular injection, which may result in local anaesthetic toxicity and intraneural injection which may result in nerve damage. Other complications include haematoma and failure.

Because of these potential complications, fascia iliaca blocks must be performed by trained staff members, in an area where the patient can be adequately monitored both during and post-procedure [23].

A femoral nerve block can be used as an alternative to fascia iliaca block. Nerve-stimulator-guided femoral nerve block has been found to provide superior analgesia when compared to fascia-iliaca block performed using the landmark technique [24]. However, as nerve-stimulator-guided femoral nerve block requires a higher skill level, is more expensive and, even in experienced hands, takes much longer to perform, fascia iliaca blocks are usually the preferred technique.

Epidural analgesia can be provided to patients on hospital admission. One study showed this significantly reduced the number of cardiac events in those at high risk of cardiac complications compared to intramuscular opioid [25].

However, epidural analgesia is an advanced anaesthetic technique, with many severe potential complications and requires a high level of monitoring during and post-procedure. Careful patient selection is paramount [26]. It may not be appropriate to perform an epidural in some patients who have suffered a hip fracture, for example due to anticoagulation and cardiovascular instability due to blood loss and dehydration.

Other options for pre-operative analgesia include mechanical techniques, such as skin traction. Unfortunately, these have not been demonstrated to be of benefit in hip fracture [27].

In summary, the type of pre-operative analgesia used is likely to be determined by institutional factors which balance the availability of non-specialist staff to administer a simple, potentially less effective block reliably to all patients against the availability of specialist staff to perform more effective blocks or site and monitor epidural anaesthesia.

It must be remembered that the most effective form of analgesia is surgery, which should be performed as soon as possible [28].

### 2.2 ‘Normalisation’ of the Patient

Patients with hip fractures are elderly, with multiple acute and chronic comorbidities and deciding whether or not they are ‘fit for surgery’ can be difficult.

Patients with ‘major’ medical comorbidities are more likely to develop post-operative complications than those with ‘minor’ complications [29]. However, delaying surgery for medical reasons may result in worse outcomes. Where major medical comorbidities exist, postponement of surgery without correcting these problems results in the worst outcomes, so every effort must be made to address these issues if they are the cause of delay [30].

The AAGBI guidelines clearly set out acceptable and unacceptable reasons for postponing hip fracture surgery, whilst recognising that a coordinated, multidisciplinary approach is required to address these in a timely fashion [11].

One aspect of pre-operative investigation which remains controversial is echocardiography for hip fracture patients with cardiac murmurs.

It has been shown that there is no significant increase in 30 day mortality or cardiac events in patients with hip fracture and aortic stenosis compared to those without aortic stenosis [31, 32]. However, the 2001 Confidential Enquiry into Perioperative Deaths (CEPOD] Elderly report recommended that all patients with cardiac murmurs should have pre-operative echocardiogram. It also hands the role of requesting this to the anaesthetist [33].

A retrospective analysis of 3997 hip fracture patients identified 20% as having a previously unidentified cardiac murmur, and 7% of the total group had aortic stenosis [31].

The AAGBI guidelines consider ‘awaiting echocardiography’ to be an unacceptable reason for surgical delay [11]. Aortic stenosis has important anaesthetic implications. General anaesthesia is considered to be the safest option, but some anaesthetists would also advocate the cautious use of low dose spinal or epidural anaesthesia. The presence of an experienced anaesthetist, attention to detail, the use of invasive monitoring and vasopressor drugs are all recommended in these cases [34].

### 2.3 Blood Transfusion

Patients with hip fracture may have anaemia for a variety of reasons, including pre-existing medical conditions, treatment with anticoagulant medication or perioperative haemodilution. Blood loss from the fracture site itself is often underestimated and is relatively greater with extracapsular fractures, commonly exceeding 1000ml. This may well be because the total blood loss from hip fracture is several times greater than that which is apparent at surgery [35].

Post-operative anaemia has been demonstrated to have a significant effect on several important outcomes of hip fracture care; Foss et al investigated the effect of anaemia (Hb <10) on patients following hip fracture surgery. They found a linear relationship between degree of anaemia and ability to mobilise independently, 30 day mortality and length of hospital stay [36]. Blood transfusion is however both controversial and expensive making the most appropriate threshold for transfusion a key element of study. There is a specific risk of pulmonary oedema in elderly patients with poor cardiovascular performance making aggressive transfusion undesirable. A recent Cochrane review concluded there was only limited evidence is available to support a liberal (Hb<10) instead of a restrictive (Hb <8) transfusion trigger [37]. The FOCUS study, a large international randomised multi-centre study has recently reported three year results demonstrating no difference in mortality between high-risk elderly patients treated with a liberal and those with a restrictive transfusion threshold [7].

One adjunct which may be beneficial in reducing blood loss is Tranexamic acid. This fibrin clot stabiliser was demonstrated to deliver a 10% reduction in major trauma by preventing haemorrhage [38] and its use in hip fracture has subsequently been demonstrated [39]. The authors of this study suggested that transfusion was prevented in one patient for every eight patients who received Tranexamic acid.

One other new area of scrutiny is the type and duration of packed red cell transfusion. Advantages of recently donated blood have been demonstrated in cardiac surgery [40]. A recent study on patients after hip fracture however failed to demonstrate these benefits in terms of complications or the incidence of post-transfusion delirium. Interestingly patients with delirium who were transfused packed red cells stored for less than 2 weeks following donation did demonstrate a shorter duration of post-operative delirium [41]. Further studies will be required to demonstrate whether there are truly benefits to transfusion with freshly donated blood in this patient group.

### 3 THE USE OF SCORING SYSTEMS IN PREDICTING PERIOPERATIVE MORTALITY

A number of different tools have been used to assess the perioperative risk of death following hip fracture. Such tools may be complicated, requiring application by specialist staff, and yet still provide precious little useful clinical information. There is a limited role for the use of mortality-predicting scores in providing risk-information to patients and their families as well as informing surgical or anaesthetic strategy. Perhaps the greatest use is however as a research tool to allow groups of patients to be risk-stratified and compared.

Common scores validated for use in predicting 30 day mortality in hip fracture include the Nottingham Hip Fracture Score (NHFS) [42-47], the Estimation of Physiologic Ability and Surgical Stress (E-PASS) [47], the Charlson Comorbidity Index (CCI) [48] and the Physiological and Operative Severity Score for the enUmeration of Mortality and Morbidity (POSSUM). The latter has been modified for Orthopaedic usage to the O-POSSUM [49-51].

The NHFS is becoming the most commonly used score in predicting perioperative risk for hip fracture. The score underwent a robust development process [42] and has been modified in response to validation from larger cohorts in other centres [46]. The NHFS uses seven factors which are readily available pre-operatively. These then require formulaic calculation to generate a score. The difficulty in calculation has been reduced by availability of a free smart phone application [52]. This score is now validated for mortality at 30 days [42, 43, 46], one year [45] and for early hospital discharge [44]. An evaluation of the NHFS has shown it to be superior compared to the other scoring systems in calculating perioperative morbidity and mortality [53].

Calculation of the POSSUM or O-POSSUM score requires 18 separate data fields combined with use of a formula. The score is biased towards the physiological state of the patient using bedside observations and blood parameters then matching this with the type of surgery which is expected. The score performed well in validation studies, but the main disadvantage of the POSSUM score is laborious input of the necessary data and difficulty in calculation of the score [59, 51]. It has also been found to overestimate mortality in hip fracture patients [51].

The CCI is calculated from 10 fields with simple addition of the score. The score is biased towards the comorbid state of the patient, requiring details of different comorbidities. The advantage of this simple score is that it can easily be calculated, and in the UK this information is collected as part of the Hospital Episode Statistics (HES) data on every patient receiving treatment. This allows the score to be applied to large cohorts of patients. The accuracy of the score is however attenuated by variation in how each comorbidity is defined and by the high rates of undiagnosed comorbidity in the hip fracture population [48].

Despite comprehensive validation of scores such as the NHFS, such tools still fail to play a material role in clinical decision-making in this patient population.

### 3.1 Intra-Operative Management

The main options for anaesthetising of a patient with hip fracture are either general or regional anaesthesia, or a combination of both.

Recent research has focused on trying to determine whether general or regional anaesthesia is advantageous in terms of reducing mortality and postoperative complications, but neither has been consistently shown to be superior [36, 54-59].

### 3.2 General Anaesthesia

This involves administering drugs, *via* either the inhalational or intravenous route, or a combination of both, to achieve a state in which the patient is unconscious and will not respond to the surgical stimulus. This will necessitate some level of airway management, either a supraglottic device, such as a laryngeal mask airway (LMA) or endotracheal intubation. General anaesthesia is commonly combined with other post-operative analgesic techniques such as nerve blocks, intravenous opiates or occasionally regional anaesthesia.

### 3.3 Regional Anaesthesia - Central Techniques

Spinal anaesthetic involves injection of local anaesthetic into the cerebrospinal fluid. The addition of an opioid extends the duration of the block and provides prolonged analgesia. Although surgery can safely proceed under spinal anaesthetic alone, most anaesthetists may also administer some sedation to improve patient experience.

Potential benefits of spinal anaesthesia include a reduction in the amount of parenteral opiates, helping to reduce post-operative confusion, a potential reduction in thromboembolism and as a safer option for patients with significant respiratory disease. Relative contraindications include aortic stenosis and anticoagulant therapy. Patient refusal is an absolute contraindication, but rare if the procedure and its benefits are explained properly.

The AAGBI guidelines recommend the use of hyperbaric or ‘heavy’ bupivacaine and positioning the patient laterally with the fractured hip inferior. This position, and the addition of tilting the table so that the caudal extremities of the patient are closer to the ground and more dependent, has a dual effect of firstly causing a profound sensory block to the fractured hip and secondly of reducing the extent of the sympathetic blockade resulting from unwanted cephalad and contralateral spread of the spinal anaesthetic [60]. This helps minimize the risk of hypotension.

The use of a catheter inserted into the epidural space is another option for intraoperative anaesthesia and analgesia. This has a much slower onset than spinal anaesthesia, which may be beneficial in patients with cardiovascular instability.

Post-operative epidural anaesthesia has been demonstrated to reduce opiate requirements during rehabilitation and provide effective dynamic analgesia without impairing motor function [61].

Combined spinal epidural techniques can also be used, providing a rapid onset of anaesthesia and analgesia from the spinal anaesthetic with epidural analgesia which can be used intra-operatively if required, or solely for post-operative analgesia.

All three of these central techniques require careful patient selection and the risks and benefits must be explained to the patient in order for informed consent to be obtained.

### 3.4 Regional Anaesthesia - Peripheral Techniques

These should be considered regardless of the mode of anaesthesia [9, 11].

Fascia iliaca and femoral nerve blocks are most commonly performed, with or without catheter insertion for continuous infusion of local anaesthetic. Alternatives include 3-in-1 and lumbar plexus blocks.

The UK 2014 SPRINT Audit (ASAP) was conducted during a three-month period. It aimed to investigate which anaesthetic techniques were being used in the perioperative care of hip fracture patients. The results showed that 50.7% of patients had a general anaesthetic, 44% had a spinal anaesthetic, 3.4% had both a general and spinal anaesthetic and 0.2% had an epidural [1]. This is very similar to data from 2009, when 51% received general and 49% regional anaesthesia [62]. ASAP reported 56% of patients received an intraoperative nerve block, 55.9% were fascia iliaca blocks and 26.6% femoral nerve block’ compared to only 19% in 2009.

### 3.5 Intra-Operative Hypotension

Avoiding intraoperative hypotension is important, as it has been shown to be associated with increased five and thirty day mortality [57].

General anaesthesia is associated with more hypotension than spinal anaesthesia, with 85.2% of patients having a systolic blood pressure < 100 mmHg intraoperatively. Combining spinal and general anaesthesia increased this risk further, to 92.2% [1].

The volume of spinal anaesthetic has been correlated with the degree of hypotension, with volumes > 1.5ml of 0.5% hyperbaric bupivacaine causing significantly more episodes than <1.5ml [63]. The ASAP found a median dose of 2.5ml was used. Absolute hypotension (SBP <100mmHg) occurred in 66.3% of patients who received spinal anaesthesia [1].

Other anaesthetic techniques used to avoid intra-operative hypotension include goal-directed fluid therapy. Invasive monitoring, such as intra-arterial cannulae, central venous catheters or oesophageal doppler probes are used to guide fluid administration according to pre-determined protocols. However, this technique has not been shown to be beneficial in patients undergoing hip fracture surgery [64, 65].

Regardless of the anaesthetic technique employed, hypotension should be avoided. Strategies for this include pre-operative fluid resuscitation, low dose spinal anaesthesia and avoiding combined general and spinal anaesthesia.

An important differential diagnosis to consider if intra-operative hypotension occurs is Bone Cement Implantation Syndrome (BCIS). This term describes cardiovascular compromise happening when cement is used part of the operative procedure. It can cause significant perioperative morbidity and mortality with clinical manifestations ranging from grade 1: moderate hypoxia (SpO2 <94%) or reduction in systolic blood pressure <20%, through to grade 3: cardiovascular collapse requiring cardiopulmonary resuscitation [66].

The ASAP study found the incidence to be 19%. Grade 2 occurred in 2.7% of cases and grade 3 in 0.5%. As a result of these findings, new safety guidelines were published in 2015 [67]. The recommendations involve a multidisciplinary approach to prevention and management of BCIS;

- Pre-operative identification of patients at high risk of BCIS and allocation of roles in case of a severe reaction.
- Intra-operative procedures to be followed by the surgeon.
- Information for anaesthetists to aid recognition of BCIS and how to manage it should it arise.

Pre-operative identification of patients at high risk of BCIS should prompt consideration of invasive monitoring and careful attention to volume status prior to cement insertion.

### 3.6 Post-Operative Management

In the post-operative period, attention should be paid to ensuring adequate oxygenation and analgesia in order to prevent complications such as chest infections, delirium and pressure sores and allow mobilisation. Venous thromboprophylaxis and fluid and electrolyte management are also a priority [11].

Pain scores are usually low following hip fracture surgery, although dynamic pain may be higher with dynamic hip screw or intramedullary nailing [68].

If a femoral nerve or fascia iliaca catheter has been inserted this is usually removed the day after surgery, depending on local protocols. If pain persists post-operatively, these peripheral blocks can be repeated once other causes of pain of have been excluded. Otherwise pain should be managed with regular paracetamol, avoiding non-steroidal anti-inflammatories and cautious use of opioids [9, 11].

It may be appropriate for high risk patients to be managed in a High Dependency Unit post-operatively [69], however the emphasis should be on physiotherapy and early mobilisation to facilitate rehabilitation and discharge [9].

## CONCLUSION

Hip fracture anaesthesia is evolving in response to a growing evidence-base regarding the perioperative care of these patients. Timely surgery is associated with improved outcomes.

Consideration should be made to performing peripheral nerve blocks pre-operatively to avoid the adverse effects of opioids. Both general and spinal anaesthesia should be supplemented with peripheral nerve blockade and intra-operative hypotension should be avoided regardless of mode of anaesthesia.

Developments in hip fracture anaesthesia will only be successful as part of a coordinated system of care for this frail patient group.

## LIST OF ABBREVIATIONS

NHFD: = National Hip Fracture Database
AAGB: = Association of Anaesthetists of Great Britain and Ireland
CEPOD: = Confidential Enquiry into Perioperative Deaths
FOCUS: = Functional Outcomes in Cardiovascular Patients Undergoing Surgical Hip Fracture Repair
NHFS: = Nottingham Hip Fracture Score
EPASS: = Estimation of Physiologic Ability and Stress Score
CCI: = Charlson Comorbidity Index
POSSUM: = Physiological Operative Severity Score for enUmeration of mortality and Morbidity
OPOSSUM -: = Orthopaedic Physiological Operative Severity Score for enUmeration of mortality and Morbidity
HES: = Hospital Episode Statistics
LMA: = Laryngeal Mask Airway
ASAP: = Anaesthetic Sprint Audit of Practice
BCIS: = Bone Cement Implantation Syndrome
